# Suppression of Jasmonic Acid-Dependent Defense in Cotton Plant by the Mealybug *Phenacoccus solenopsis*

**DOI:** 10.1371/journal.pone.0022378

**Published:** 2011-07-27

**Authors:** Pengjun Zhang, Xiaoyun Zhu, Fang Huang, Yong Liu, Jinming Zhang, Yaobin Lu, Yongming Ruan

**Affiliations:** 1 State Key Laboratory Breeding Base for Zhejiang Sustainable Pest and Disease Control, Institute of Plant Protection and Microbiology, Zhejiang Academy of Agricultural Sciences, Hangzhou, China; 2 Department of Plant Protection, Nanjing Agriculture University, Nanjing, China; 3 College of Chemistry and Life Sciences, Zhejiang Normal University, Jinhua, China; University of California, United States of America

## Abstract

The solenopsis mealybug, *Phenacoccus solenopsis*, has been recently recognized as an aggressively invasive pest in China, and is now becoming a serious threat to the cotton industry in the country. Thus, it is necessary to investigate the molecular mechanisms employed by cotton for defending against *P. solenopsis* before the pest populations reach epidemic levels. Here, we examined the effects of exogenous jasmonic acid (JA), salicylic acid (SA), and herbivory treatments on feeding behavior and on development of female *P. solenopsis*. Further, we compared the volatile emissions of cotton plants upon JA, SA, and herbivory treatments, as well as the time-related changes in gossypol production and defense-related genes. Female adult *P. solenopsis* were repelled by leaves from JA-treated plant, but were not repelled by leaves from SA-treated plants. In contrast, females were attracted by leaves from plants pre-infested by *P. solenopsis*. The diverse feeding responses by *P. solenopsis* were due to the difference in volatile emission of plants from different treatments. Furthermore, we show that JA-treated plants slowed *P. solenopsis* development, but plants pre-infested by *P. solenopsis* accelerated its development. We also show that *P. solenopsis* feeding inhibited the JA-regulated gossypol production, and prevented the induction of JA-related genes. We conclude that *P. solenopsis* is able to prevent the activation of JA-dependent defenses associated with basal resistance to mealybugs.

## Introduction

The solenopsis mealybug, *Phenacoccus solenopsis* Tinsley (Hemiptera: Pseudococcidae), was described originally from U.S.A in 1898 [1], suggesting it is native there. This exotic insect pest has been spreading through out the entire American region since it was first reported in Mexico in 1978. Since then, it has spread to South American from 1985 onwards and Central American from 1986 onwards [2]. *P. solenopsis* has spread to Asia, where it was first reported in Pakistan in 2005 [3]; and has rapidly spread to other countries, including India, Thailand, and Australia [4], [5], [6]. Recently, *P. solenopsis* was reported for the first time in China, in Guangzhou on *Hibiscus rosa-sinensis* [7].

Mealybug *P. solenopsis* feeds on numerous crops, weeds, ornamentals and medical plants, and its adults and nymphs are able to inflict severe damage on leaves, fruit, main stems and branches by feeding on phloem sap and egesting sugary honeydew [5]. Cotton (*Gossypium hirsutum* L.) is one of the most favored host plants for *P. solenopsis*, and both Bt- and non-Bt cultivars of cotton can be fed by the mealybug [8]. *P. solenopsis* has been reported to severely affect the cotton industry in Pakistan and India. In the cotton producing area of Pakistan (totaling 8.0 million acres), over 150,000 acres has been seriously damaged by *P. solenopsis* [9]. In India, *P. solenopsis* infestation was recorded on *G. hirsutum* from nine cotton-growing states in 2006 [10]. Recently, *P. solenopsis* has been recognized as an aggressively invasive species on agricultural and ornamental plants in China [11]. If *P. solenopsis* cannot be controlled in all these areas, Wang et al. [12] forecasted that the losses in cotton yield in 2008/2009 would be 1.4 million tons in China, 1.12 million tons in India and 0.48 million tons in Pakistan. However, the molecular mechanism of cotton in response to *P. solenopsis* feeding has not been investigated to date. A basic understanding of the mechanisms of cotton resistance to the mealybugs will provide a new insight into how the eruption of mealybugs occurred and how to develop more durable resistance.

Like aphids and whiteflies, *P. solenopsis* is an obligate phloem-feeding pest. These species are known for their “stealthy” feeding mechanisms that cause minimal damage to plant tissues as they establish direct nutritional access through the vascular tissue. To date, most studies of phloem-feeding insects have focused on the interactions of aphids or whiteflies with their host plants [13]–[17]. Previous studies of plant response to phloem-feeding insects suggest that jasmonic acid (JA)-, salicylic acid (SA)-, and ethylene-dependent signaling pathways were at least partially activated by phloem-feeding insects. A lipoxygenase, which is a key enzyme in JA synthesis in plants and induced by wounding [18], was up-regulated in several plant-aphid interactions, including in tomato to potato aphid (*Macrosiphum euphorbiae*) and green peach aphid (*Myzus persicae*) [19], sorghum to the greenbug aphid [14], and Arabidopsis to the green peach aphid [13]. In contrast, the SA-dependent *PR* genes were both locally and systemically induced by aphid or whitefly feeding in tomato and Arabidopsis [13], [15], [16]. Direct quantification has also demonstrated that aphid or whitefly induce SA accumulation in many plants [20], [21].

However, the impact of SA or JA defense pathway on resistance to phloem-feeding insect remains controversial [22], [23]. In tomato, basal SA defenses decrease *M. euphorbiae* longevity, and SA is important in *Mi1*-mediated resistance potato aphid [24]. In Arabidopsis, Pegadaraju et al. [23] observed a decrease in aphid numbers on two mutant lines that have elevated SA levels (*cpr5* and *ssi2*) and an increase in aphid populations on a mutant that has reduced SA accumulation (*pad4*). A small number of studies, however, have shown that artificial induction JA-regulated defense has a negative impact on phloem-feeding insects [16], [25]–[27]. Considering that *P. solenopsis* is a phloem-feeing insect, it is necessary to address the following questions: 1) whether JA- or SA-regulated defense play a key role in basal resistance to *P. solenopsis*; 2) how the *P. solenopsis* cope with plant defense responses?

In this study, we examined the effects of exogenous JA, SA, and herbivory treatments of cotton on adult feeding choice and development of *P. solenopsis*. We also compared the volatile emissions of cotton plants upon JA, SA, and herbivory treatments, as well as the time-related changes in gossypol production and defense-related genes. We show that JA-dependent defenses play a key role in basal resistance to *P. solenopsis*. However, *P. solenopsis* could inhibit JA-dependent responses in cotton through its feeding behavior. The manipulation of plant defenses (the “decoy” hypothesis) by *P. solenopsis* to enhance its performance may have contributed to the rapid invasion of *P. solenopsis* in China and elsewhere.

## Materials and Methods

### Insects and Plants

Mealybugs, *Phenacoccus solenopsis* Tinsley (Hemiptera: Pseudococcidae), were originally collected from *Hibiscus rosa-sinensis* in Hangzhou (30°10′N, 120°15′E), China, and maintained on potted plants of the cotton, *Gossypium hirsutum*, cultivar Zhefengmian No. 1, in a climate controlled room (26±2°C, 65–75% RH, 14L∶10D photoperiod).

Cotton plants (*G. hirsutum*, cv. Zhefengmian No. 1) were grown in an insecticide-free greenhouse compartment under natural light and 30/25°C temperature. All plants were used in experiments at the 3–4 fully expanded true leaf stage, which occurred six weeks after sowing.

### Plant Treatment

1) JA or SA treated plants: JA or SA (Sigma-Aldrich) was dissolved in 1 mL of acetone and dispersed in water (containing 0.1% Tween 20) to produce three concentrations of JA or SA solution: 1 mM, 0.1 mM, and 0.01 mM. We liberally sprayed the foliage of each plant with 1.0 mL/leaf of JA or SA solution with a hand-sprayer. Treated plants were used in the feeding-choice tests and volatile trapping experiments 24 h after JA or SA application. For gene-expression bioassays, leaf samples were collected 1d, 3d, and 5d after JA or SA application.

2) Herbivore-damaged plant: A mixture of third instar nymphs and young adults (totaling 50) of *P. solenopsis* were carefully transferred using soft brush onto each plant, and allowed them to feed freely on plant for 1d, 3d, 5d, and 7d. After that, leaf samples were collected for gene-expression bioassay. Plants pre-infested by mealybugs for 5 days were used for feeding choice tests and volatile trapping experiments.

3) Intact control plants: Intact plants were sprayed with 1.0 mL/leaf of water (containing 0.1% Tween 20) and were used these plants as controls for comparison with JA- or SA- treated plants. Intact plants, that received no treatment, were used as controls for comparison with herbivore-damaged plants.

### Feeding choice tests with JA-treated, SA-treated, or mealybug-infested leaves

In this experiment, mealybugs were offered a choice of two detached leaves: one from a JA-treated, SA-treated, or mealybug-infested plant and one from a control plant. Two leaves were placed opposite each other in a Petri dish (diam 14.5 cm) covered with moist filter paper, so that they were approximately 5 cm apart at the closest point. The position of the leaves was alternated between replicates. Immediately, four new-emerged adult female mealybugs were transferred into each dish in the gap between the leaves and allowed to feed overnight. After 24 h, the number of adults on each of the two leaves was counted. The experiments were repeated for 10–15 times for each treatment.

### Chemical analysis of volatiles

Headspace volatile samples were collected as described in detail by Zhang et al. [28]. Samples analyses were carried out with a Shimadzu GC-2010 plus GC-MS (Shimadzu, Japan) equipped with an Rxi-5MS (30 m-0.32 mm i.d., 0.25 µm film thickness) column. The column effluent was ionized by electron impact ionization (70 eV). Mass scanning was done from 33 to 250 *m*/*z*. The temperature programs of the GC were as follows: 40°C (4-min hold), 8°C min^−1^ to 250°C (5-min hold). Compounds were identified by comparing the mass spectra with those of authentic standards or with NIST 08 spectra. Quantification of identified compounds was based on comparison with a set of authentic compounds injected in different concentrations ranging from 2.5 ng to 20 ng/µL methanol. Response factors were linear for all reference compounds within this concentration range.

### Performance on JA-treated, SA-treated, or mealybug-infested plants

To determine the effects of JA, SA, and herbivory treatments on the performance of *P. solenopsis*, development time and adult weight gain of female *P. solenopsis* were assayed. Five young eggs (≤24 h) from the same cohort were transferred onto a detached leaf from JA-treated, SA-treated, and mealybug-infested plants, respectively. After that, each leaf with nymphs was individually placed in a ventilated Petri dish (diam 9.0 cm), and its petiole was covered with moist cotton wool to keep the leaf fresh. The nymphs were reared in a climate-controlled room (26±2°C, 65–75% RH, 14L∶ 10D photoperiod). Male nymphs were excluded from the population. Observations were made twice daily on survival, development and feeding until adults emerged. The development time from egg to adult of each female mealybug was recorded. Leaves were replaced every four days over the period of 20 days. The experiment was replicated 20 times for each treatment.

Another 60 young nymphs (≤24 h) hatching from the same cohort of eggs were reared on cotton plants until they attained adulthood. A newly-emerged adult female was weighed by using a Shimadzu AY220 electronic balance, and individually transferred onto a detached leaf from JA-treated, SA-treated, and mealybug-infested plants, respectively. Each leaf with the females was placed in a ventilated Petri dish (diam 9.0 cm) as described above. Twenty females were tested for each treatment. They were maintained in the climate controlled room for ten consecutive days. Following that, adult females were weighed again.

### Gossypol extraction and HPLC analysis

The ground, lyophilised leave samples (100 mg) were weighed into centrifuge tubes and extracted by ultrasonification (3 min) in solvent 1 (acetonitrile/water/phosphoric acid = 80∶20∶0.1; 10 mL). The samples were centrifuged (3 min at 2800 *g*), and an aliquot of the supernatant was transferred directly into an autosampler vial. Standard gossypol (95% purity; Sigma-Aldrich) was dissolved in solvent 1. Standard curves were obtained for gossypol with concentrations in the range of 5–80 µg mL^−1^ in 5 increments. Three samples were collected for each treatment.

Samples were analyzed on a Waters 2695 high-performance liquid chromatograph equipped with a UV-VIS detector (Waters 2489). Samples were isocratically eluted from a 150×3.9 mm i.d. Waters (4 µm) C18 Novapak column maintained at 40°C. The mobile phase was the same as that used by Stipanovic et al. [29] and was helium purged. Solvent flow rate was 1.0 mL min^−1^ and total run time was 30 min. The signal was monitored at 272 nm. Data collection and integration were performed using the Waters Empower software.

### Total RNA isolation and cDNA synthesis

To minimize wounding- and dehydration-induced gene expression, leaf samples were quickly harvested and immediately frozen in liquid nitrogen. For each sample, tissues from three plants were pooled. Frozen samples were ground to a fine powder in liquid nitrogen with a pestle and mortar. Total RNA was extracted from 150 mg of each leaf sample using a plant RNA isolation kit (Axygen, Hangzhou, China), according to the manufacturer's instructions. RNA concentration and purity were determined using a NanoDropTM Spectrophotometer ND-2000 (Thermo Scientific, Wilmington, USA), and the integrity of RNA was also assessed by 1% agarose gel electrophoresis and ethidium bromide staining. The presence of contaminant DNA in the RNA samples was verified by PCR using specific primers of a known gene (e.g. *GhACT4*) and gel electrophoresis analysis. No fragments of genomic DNA were identified in all samples tested in this work (data not shown). First stand cDNA was synthesized from 200 ng RNA using a First-Strand cDNA Synthesis Kit (TaKaRa, Hangzhou, China) according to the manufacturer's instructions.

### Real-Time PCR

To quantify *lipoxgenase* (*GhLOX1*), *β-1,3-glucanase*, and *acidic chitinase* transcript levels in different samples, real-time quantitative RT-PCR was performed. The real-time PCR was carried out on an ABI 7500 Real Time PCP System (Applied Biosystems) with a 96 well rotor. The amplification reactions were performed in 20 µl final volume containing 10 µl of SYBR® Premix Ex Taq™ (TaKaRa, Hangzhou, China), 0.8 µl of forward primer (5 µM) and reverse primer (5 µM) pairs and 2 µl cDNA first strand template. Thermal cycling conditions were 5 min at 95°C, followed by 35 cycles of 15 s at 95°C, 15 s at 55°C and 30 s at 72°C. Subsequently, melting curve was recorded between 60°C and 95°C with the hold every 5 s. All reactions were run in duplicate. The melt-curve analysis ensured that the resulting fluorescence originated from a single PCR product and did not represent primer dimers formed during the PCR or due to non-specific product. No-template control as water and minus RT (10 ng of RNA) were also included to detect any spurious signals arising from amplification of any DNA contamination or primer dimer formed during the reaction. The *GhACT4* was used as a housekeeping gene as its expression was most stable in cotton leaves [30]. Primers used for quantitative RT-PCR are given in Table 1. The relative gene expression was calculated using the comparative 2^−ΔΔ*Ct*^ method with *GhACT4* as endogenous control gene [31].

**Table 1:** Primer sequences used for qPCR analysis.

Gene	Forword primer (5′-3′)	Reverse primer (5′-3′)
*GhACT4*	TTGCAGACCGTATGAGCAAG	ATCCTCCGATCCAGACACTG
*GhLOX1*	ACATGCCGAAGCCGCTGCTT	GGGCGTATTCGGGGCCCTTG
*β-1,3-glucanase*	AATGCGCTCTATGATCCG	GATGATTTATCAATAGCAGCG
*acidic chitinase*	GCTCAGAATTCCCATGAAACTACAGGTG	GGTTGGATCCTTTGCGACATTC

### Statistical Analysis

A replicated *G*-test of goodness-of-fit was used to analyze the feeding choice of *P. solenopsis* between treated and control leaves, with the null hypothesis of no preference. Females that did not make a choice were excluded from the analysis. Fisher's protected least significant difference (PLSD) test of ANOVA was used to analyze the data of development time and adult weight. The data of gene expression were log-transformed and statistically analyzed by a one-way ANOVA. Gossypol data from different plant treatments was analyzed by ANOVA followed by Fisher's LSD multiple comparison tests. The volatile patterns of differently treated plants were analyzed using principal component analysis (PCA) using SPSS (version 13.0). Fifteen major compounds were used in the PCA analysis. The amounts of volatiles collected from the different treatment plants (i.e. control plants and JA-treated plants) were compared with ANOVA followed by Tukey's HSD test for every single volatile compound separately.

## Results

### Feeding choice of mealybugs

**Figure 1:**
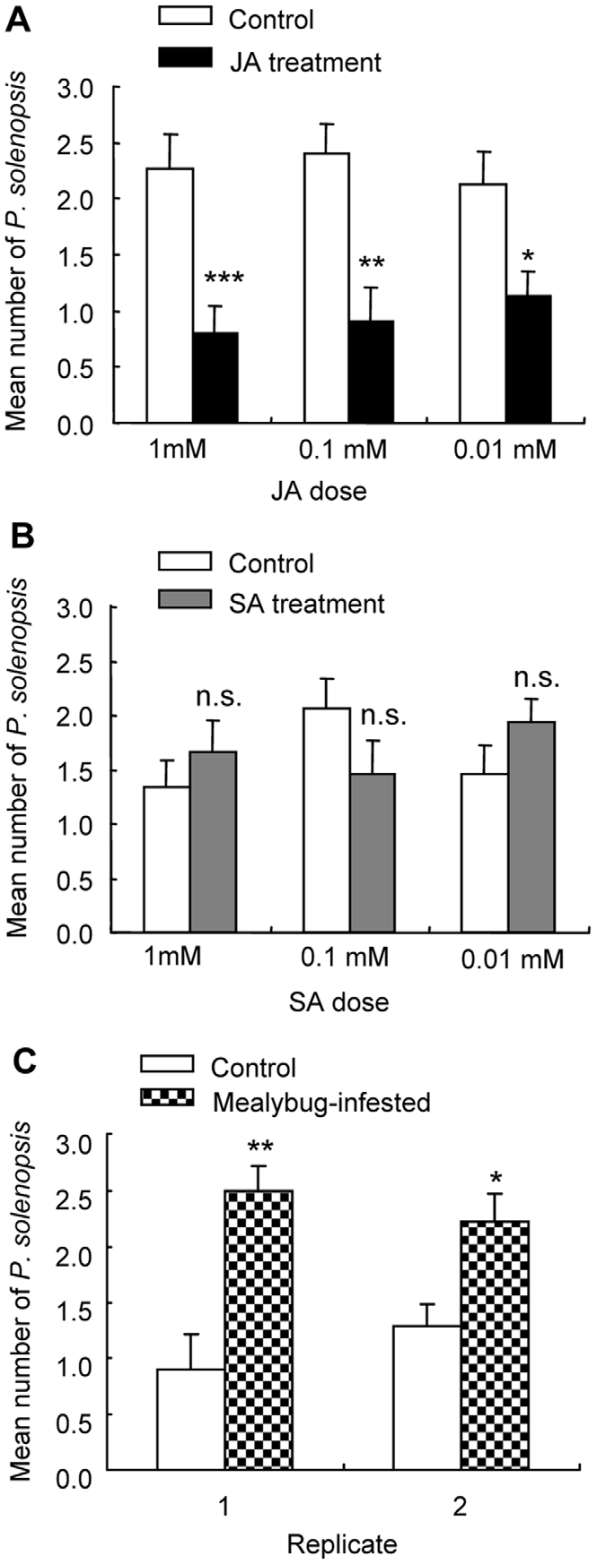
Feeding choices of *Phenacoccus solenopsis* females to JA-treated (A), SA-treated (B), or mealybug-infested leaves (C), when undamaged leaves (Control) were offered as alternative. Bars represent the mean number (± SE) of mealybugs choosing either of the plants. Asterisks represent significant differences from control plants as determined by replicated *G* test of goodness-of-fit (* P<0.05; ** P<0.01; *** P<0.001; n.s. = not significant).

When *P. solenopsis* adults were offered a choice between leaves from JA-treated and control plants, more adults chose for the leaves from control plants than for the leaves from JA-treated plants, regardless of JA dose (1 mM, *G* = 11.0, *P*<0.001; 0.1 mM, *G* = 7.1, *P* = 0.008; 0.01 mM, *G* = 4.7, *P* = 0.03; Figure 1A). When *P. solenopsis* adults were offered a choice between leaves from SA-treated and control plants, the number of adults choosing leaves from control plants did not differ from those choosing for leaves from SA-treated plants, regardless of SA dose (1 mM, *G* = 0.56, *P* = 0.46; 0.1 mM, *G* = 1.54, *P* = 0.22; 0.01 mM, *G* = 0.96, *P* = 0.33; Figure 1B). When *P. solenopsis* adults were offered a choice between leaves from *P. solenopsis*-infested and control plants, more adults chose for the leaves from *P. solenopsis*-infested plants than for the leaves from control plants (Replicate 1: *G* = 7.84, *P* = 0.005; Replicate 2: *G* = 4.32, *P* = 0.04).

### Volatile analysis

Volatile blends of control, JA-treated, SA-treated, and mealybug-infested plants were analyzed. Fifteen volatile compounds were identified (Table 2). Quantitative analysis showed that the amounts of methyl isonicotinate, methyl nicotinate, and cedrol from JA-treated plants were higher than those emitted from control plants; the amounts of β-ocimene, cyclohexane, and β-caryophyllene from SA-treated plants were higher than those emitted from control plants; the amounts of 3-henex-1-ol acetate, cyclohexane, and β-caryophyllene from *P. solenopsis*-infested plants were higher than those from control plants (Table 2).

**Table 2:** Concentration (ng·g FW^−1^·h^−1^) of volatile compounds detected in the headspace of cotton plants after different treatments.

	Compound	Control^a^ (n = 6)	Jasmonic acid (n = 5)	Salicylic acid (n = 6)	*Phenacoccus solenopsis* (n = 6)
1	α-Pinene	3.63±0.55^b^	1.78±0.78	3.50±0.64	6.59±1.56
2	α-Linalool	n.d.	0.23±0.10	n.d.	n.d.
3	3-Henex-1-ol acetate	0.43±0.27	0.25±0.12	2.10±1.00	5.81±0.97**
4	β-Ocimene	0.37±0.08	1.37±0.73	2.57±1.09*	2.30±1.12
5	β-Linalool	n.d.	n.d.	6.63±0.51	21.77±14.09
6	Methyl isonicotinate	0.13±0.13	0.91±0.39*	n.d.	n.d.
7	Methyl nicotinate	0.64±0.23	4.13±1.60*	n.d.	n.d.
8	Cyclohexane	0.81±0.31	0.68±0.22	4.05±0.44**	6.08±1.90*
9	C_25_H_50_O_2_	n.d.	0.88±0.21	n.d.	n.d.
10	C_17_H_28_O_2_	1.37±0.12	0.91±0.23	n.d.	n.d.
11	α-Cedrene	2.90±0.22	2.35±0.57	n.d.	n.d.
12	β-Caryophyllene	4.33±0.93	1.68±0.96	27.50±4.22**	58.51±29.12**
13	β-Cedrene	0.97±0.23	0.89±0.21	n.d.	n.d.
14	α-Caryophyllene	1.69±0.43	0.69±0.40	2.92±1.90	8.75±6.84
15	Cedrol	0.33±0.22	1.41±0.39*	2.22±1.03	4.23±1.65
Total amount		17.60±2.35	18.16±5.60	52.50±9.17	114.05±52.90

a Control, plants were sprayed with water 24 h before volatile trapping; Jasmonic acid, plants were sprayed with 1 mM jasmonic acid solution 24 h before volatile trapping; Salicylic acid, plants were sprayed with 1 mM salicylic acid solution 24 h before volatile trapping; *Phenacoccus solenopsis*, plants were infested with 50 of mix-aged *P. solenopsis* 5 d before volatile trapping.

b Values are means ± SE.

Asterisks indicate means of treatment significantly different from means of control (* P<0.05; ** P<0.01); n. d., not detected.

**Figure 2:**
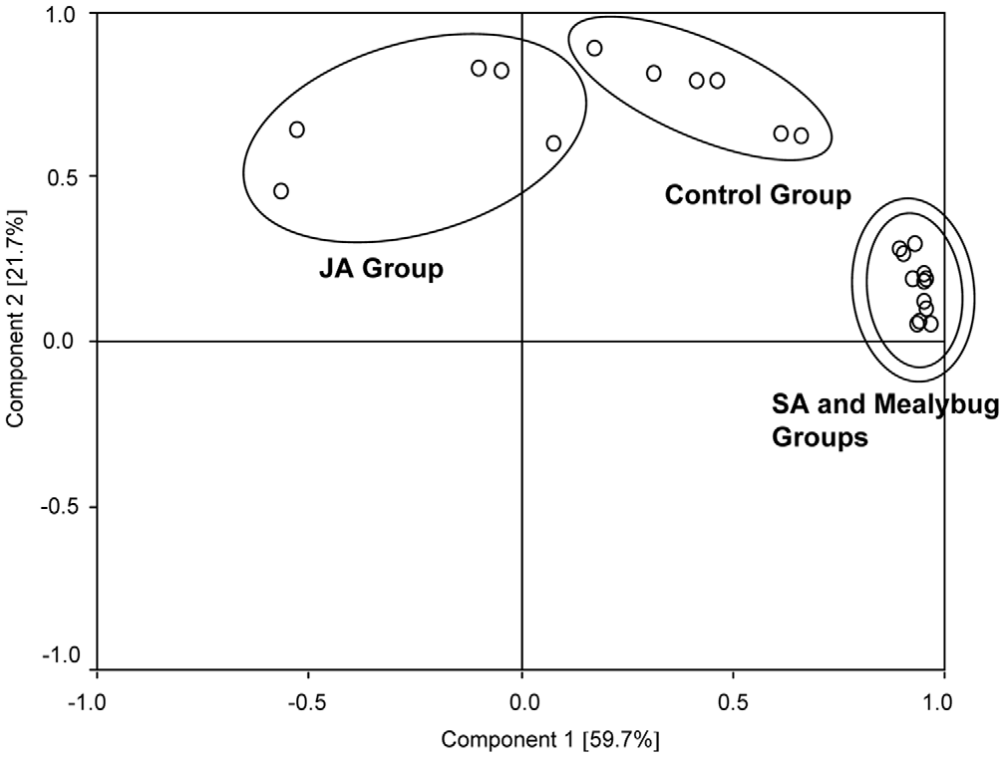
Principal component analysis (PCA) of volatile pattern of plants from different treatments. Control group, undamaged plants; Mealybug Group, plants infested with 50 of mix-aged *P. solenopsis*; JA Group, jasmonic acid-treated plants; SA Group, salicylic acid-treated plants. First and second principal component plotted against each other. Percentage variation explained between brackets.

PCA analysis showed that the volatile blend composition of *P. solenopsis*-infested and SA-treated plants highly overlapped, and both of them separated from control and JA-treated plants. The volatile blend composition of JA-treated plants separated from those of control plants (Figure 2).

### Performance of mealybugs

**Figure 3:**
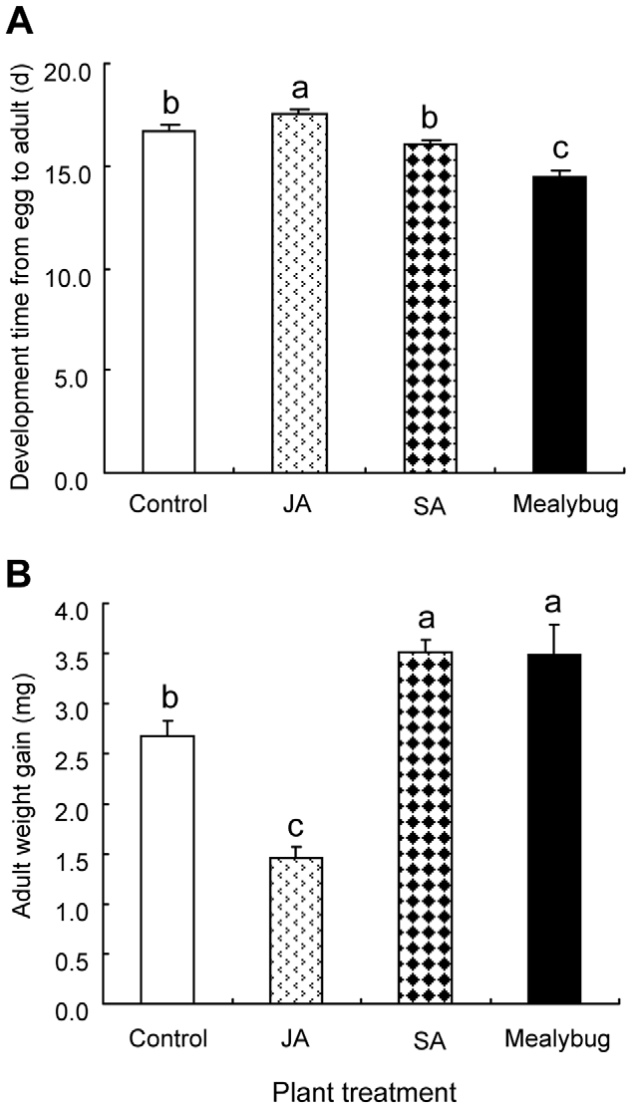
Performance of *Phenacoccus solenopsis* females reared on cotton plants from different treatments. Bars indicate means ± SE; different letters indicate significant differences in the quantities between different treatments (Fisher's PLSD test of ANOVA, P<0.05). Control, undamaged plants; JA, plants sprayed with 1 mM JA solution; SA, plants sprayed with 1 mM SA solution; Mealybug, plants pre-infested with 50 of mix-aged *P. solenopsis*.

The mean (±SE) development time from egg to adult of females reared on control leaves was 16.46±0.36 days. Compared to the development time from egg to adult of females reared on control leaves, the development time from egg to adult of females reared on *P. solenopsis*-infested leaves was significantly decreased (*P*<0.001; Figure 3A); that of females reared on JA-treated leaves was significantly increased (*P* = 0.02), but that of females reared on SA-treated leaves was not (*P* = 0.12, Figure 3A).

The mean (±SE) weight gain of adult females reared on JA-treated leaves was significantly lower than that of females reared on control leaves (*P*<0.001) (Figure 3B). In contrast, the mean (±SE) weight gains of females reared on SA-treated and *P. solenopsis*-infested leaves were significantly higher than that of females reared on control leaves, respectively (SA: *P*<0.001; Mealybug: *P*<0.001; Figure 3B).

### Gossypol analysis

**Figure 4:**
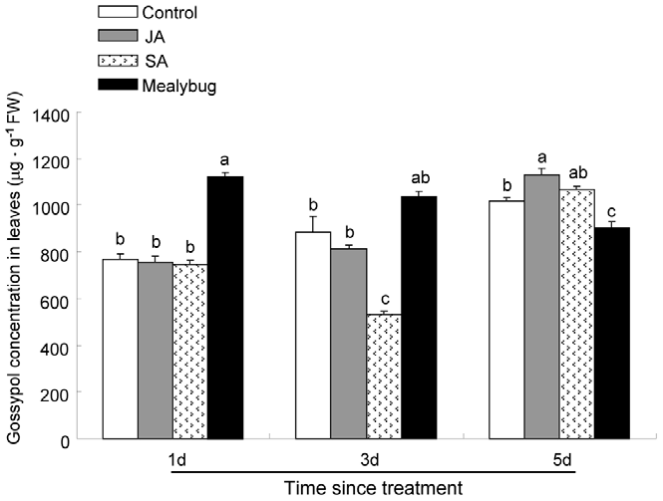
Gossypol concentration in leaves of cotton plants from different treatments. Bars indicate means ± SE of three biological replicates; significant differences among different treatments are indicated by letters on each bar. Control, undamaged plants; JA, plants sprayed with 1 mM JA solution; SA, plants sprayed with 1 mM SA solution; Mealybug, plants pre-infested with 50 of mix-aged *P. solenopsis*.

The amount of gossypol in leaves infested with *P. solenopsis* for 1d was significantly increased than that in control leaves (*P*<0.001). In contrast, after 3d of *P. solenopsis* infestation, the gossypol amount in infested-leaves did not differ from the amount in control leaves. After 5d of *P. solenopsis* infestation, the gossypol amount in infested-leaves was significantly decreased than that in control leaves (*P* = 0.015, Figure 4).

The amount of gossypol in JA-treated leaves did not differ from that in control leaves 1d and 3d after JA treatment. In contrast, 5d after JA treatment, the gossypol amount in JA-treated leaves was significantly increased than that in control leaves (*P* = 0.02, Figure 4).

The amount of gossypol in SA-treated leaves did not differ from that in control leaves 1d, 5d after SA treatment. However, 3 d after SA treatment, the gossypol amount in SA-treated leaves was significantly decreased than that in control leaves (*P*<0.001; Figure 4).

### Time-related changes in defense-related genes

**Figure 5:**
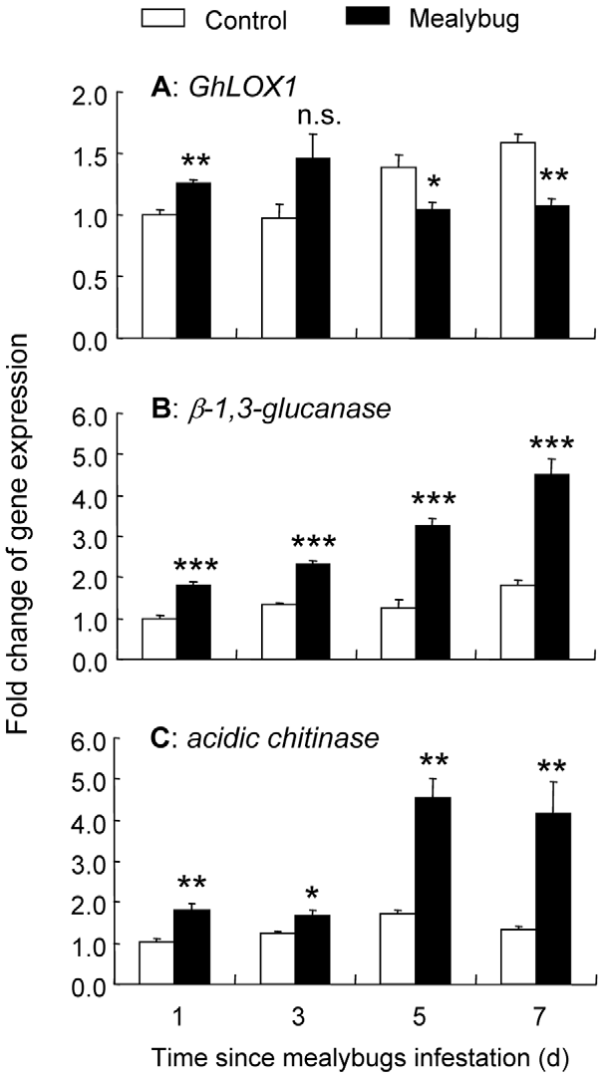
Time-related changes in expression of *GhLOX1* (A), *β-1,3-glucanase* (B), *acidic chitinase* (C) in response to mealybugs feeding in cotton. The relative gene expression was calculated using the comparative 2^−ΔΔ*Ct*^ method with *GhACT4* as endogenous control gene. Values are shown as the mean (± SE) of three biological replicates. Asterisks represent significant differences from control plants as determined by Fisher's PLSD test of ANOVA (* P<0.05; ** P<0.01; *** P<0.001; n. s. = not significant).

Using quantitative RT-PCR, we examined the time-related changes in transcript levels of one JA-dependent gene (*GhLOX1*) and two SA-dependent PR genes (*β-1,3-glucanase* and *acidic chitinase*) in plants upon JA, SA, and herbivory treatments. *GhLOX1* is mainly regulated by the JA-dependent signaling pathway in cotton [32]. *β-1,3-glucanase* and *acidic chitinase* are known as two pathogenesis-related genes, which are mainly regulated by the SA-dependent signaling pathway [33], [34]. In response to mealybug feeding, *GhLOX1* transcript levels were significantly increased at 1d after *P. solenopsis* feeding, but not at 3d. In contrast, *GhLOX1* transcript levels were significantly decreased at 5d and 7d after *P. solenopsis* feeding (Figure 5A). *β-1,3-glucanase* transcript levels were consecutively induced for 7d, with maximum induction at 7d (Figure 5B). Similarly, *acidic chitinase* transcript levels were consecutively induced for 7d, with maximum induction at 5d (Figure 5C).

**Figure 6:**
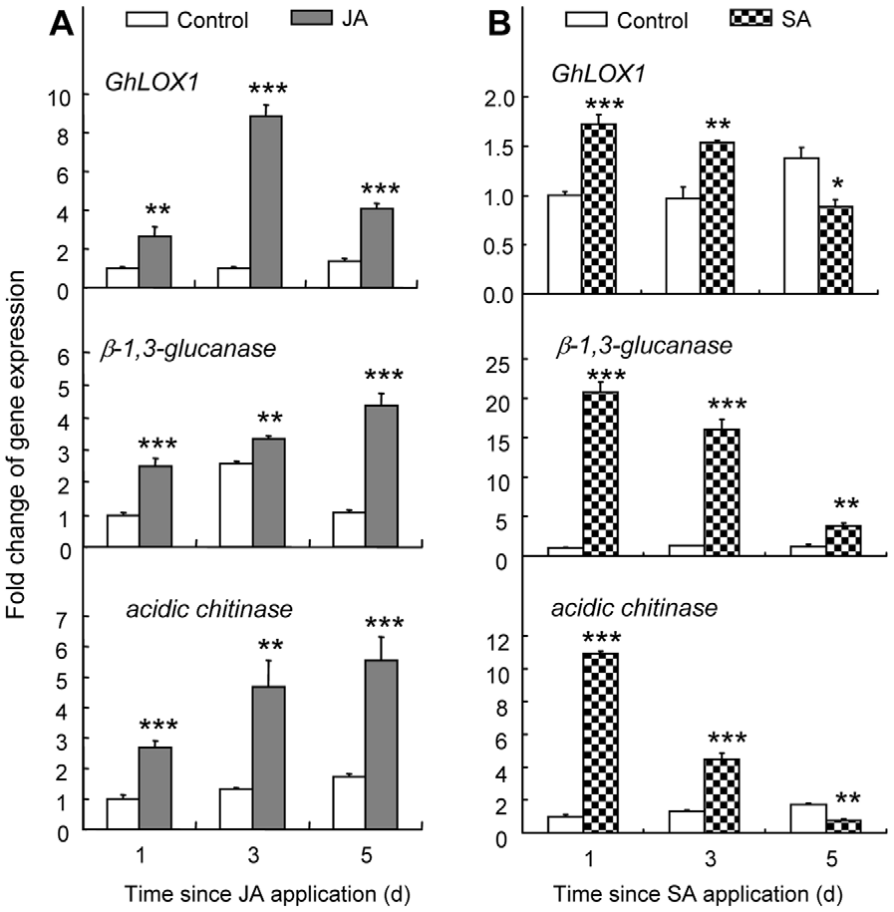
Time-related changes in expression of *GhLOX1*, *β-1,3-glucanase*, *acidic chitinase* in response to JA (A) or SA (B) treatment in cotton. The relative gene expression was calculated using the comparative 2^−ΔΔ*Ct*^ method with *GhACT4* as endogenous control gene. Values are shown as the mean (± SE) of three biological replicates. Asterisks represent significant differences from control plants as determined by Fisher's PLSD test of ANOVA (* P<0.05; ** P<0.01; *** P<0.001; n. s. = not significant).

In response to JA treatment, *GhLOX1* transcript level was significantly induced during the 5d-experimental period, and reached a peak at 3d (Figure 6A). However, it should be noted that JA also consecutively induced the two SA-dependent genes, *β-1,3-glucanase* and *acidic chitinase* (Figure 6A), suggesting that the two pathways are not always exclusive.

In response to SA treatment, *GhLOX1* transcript levels were significantly induced 1d and 3d after SA treatment, but significantly decreased 5d after SA treatment (Figure 6B). *β-1,3-glucanase* transcript levels were consecutively induced for 5d, with maximum induction at 1d (Figure 6B). *Acidic chitinase* transcript levels were induced 1d and 3d after SA treatment, but decreased 5d after SA treatment (Figure 6B).

## Discussion

The mealybug *P. solenopsis* is an invasive insect pest that is new to China. Since the mealybug was first reported in China in 2008 [7], it has rapidly spread throughout South China causing serious economic losses on cotton production [12]. Other than the field survey on its damage on cotton, and determining its morphology and environmental adaptability [4], [5], [10], [11], there has been little research done on the molecular response to *P. solenopsis* feeding in cotton plants.

This is the first report to investigate the effectual defense response to the *P. solenopsis* in cotton, and to show the induction of defense-related genes of multiple plant response pathways by *P. solenopsis* feeding on leaves of cotton. Our results demonstrate that JA-treated plants resulted in a significant repellency to adult *P. solenopsis*, which was due to the induction in emission of methyl nicotinate and cedrol from JA-treated plants (Figure S1). Moreover, female *P. solenopsis* developed slower and achieved a lower adult weight through feeding on JA-treated plants. In contrast, SA-treated plants did not show repellency to adult *P. solenopsis*; and SA treatment had no effects on nymphal development, but significantly increased adult weight. These data suggest that JA-dependent but not SA-dependent defense pathways may be involved in basal defense against *P. solenopsis* in cotton.

Jasmonic acid is known as an essential component in the octadecanoid pathway, involved not only in induced direct defense against herbivores in plants, but also in induced indirect defense [35]–[37]. However, only a few studies have documented the role of JA signaling pathway in defending against phloem-feeding insects. Ellis *et al.* [27] found that *M. persicae* grew less well on the mutant *cev-1*, which has enhanced JA signaling, than on wild-type Arabidopsis plants, and grew better on *coi1-16*, with impaired JA signaling. Likewise, *Bemisia tabaci* nymphal development was significantly delayed when reared on the Arabidopsis mutant *cev*-*1* that activates JA defense [16]. Mewis et al. [22] reported that the negative effects of JA-dependent defense on phloem-feeding insect development could be related to the induction of glucosinolate (GS) by JA. Populations of two aphids, *M. persicae* and *Brevicoryne brassicae*, were negatively correlated with constitutive and induced GS levels in Arabidopsis; and both aphid species performed better on the mutant *coi1*, in which JA signaling is blocked and constitutive GS level are low [22].

While JA-dependent responses play an important role in defense against phloem-feeding insects, there is growing evidence that phloem-feeding insects could inhibit the JA-signaling pathway through their feeding. For instance, JA-regulated genes are induced transiently or at lower levels following aphids feeding in many plants [13], [14], [38]. Further, JA-dependent genes, such as *PDF1.2* and *VSP1*, were significantly repressed upon *B. tabaci* feeding in Arabidopsis [15], [16]. Likewise, our data also demonstrated that *P. solenopsis* was able to evade eliciting the JA signaling pathway in cotton. *P. solenopsis* feeding inhibited the JA-regulated gossypol production, but also suppressed the expression of a JA-regulated gene (*GhLOX1*). Furthermore, *P. solenopsis* accelerated itself development when feeding on plants pre-infested by conspecifics, which provided behavioral evidence for inhibiting JA-regulated defense by *P. solenopsis*.

In contrast to the suppression of JA-dependent responses, *P. solenopsis* feeding induced similar responses as those induced by SA treatments, including volatile patterns and suppression of gossypol production. Moreover, *P. solenopsis* feeding strongly induced expression of two SA-regulated genes, *β-1,3-glucanase* and *acidic chitinase* [33], [34]. These results indicated that plant responses to *P. solenopsis* feeding were SA-dependent in cotton. This result is consistent with previous findings on the induction of SA signaling pathway by other phloem-feeding insects. For example, the SA-dependent *PR* genes are mainly induced by aphid or whitefly feeding in tomato and Arabidopsis [13], [15], [16]. Also, aphid or whitefly feeding induces the accumulation of endogenous SA in many plants [20], [21].

As respect to the underlying mechanisms for suppression of JA-dependent defense by *P. solenopsis*, we speculated that there were two possibilities. First, strong induction of SA-dependent responses by *P. solenopsis* may suppress the JA-signaling pathway due to cross-talk between the two pathways [39], [42]. Second, *P. solenopsis* could prevent the activation of JA defense by introducing inhibitors that directly or indirectly antagonize JA-signaling pathway. And these inhibitors are assumed to be a salivary component synthesized by phloem-feeding insects or its endosymbionts [40]. However, a recent study showed that additional *B. tabaci* feeding inhibited the accumulation of endogenous JA and also endogenous SA induced by spider mites (Tetranychidae: Acarina) in Lima bean, *Phaseolus lunatus* [21], which suggested that the suppression of JA signaling pathway by whiteflies could be due to other phytohormones. Further experiments that examine the cross-talk with transgenic cotton mutants and *P. solenopsis* salivary components will allow identification of the mechanisms responsible for suppression of the JA-dependent pathway by *P. solenopsis* feeding.

It should be noted that *P. solenopsis* preferred to conspecific-infested plants, and also achieved significant benefits through feeding on infested plants. These data suggested that *P. solenopsis* feeding compromised JA-regulated basal resistance to mealybugs. Furthermore, *P. solenopsis* feeding mainly induced the SA-dependent responses, but inhibited the JA-dependent responses. The correlation between defense responses induced by *P. solenopsis* and their performance on JA- and SA-treated plants show that *P. solenopsis* could manipulate plant signaling to suppress effective defenses, and enhance its performance. This is consistent with the “decoy” hypothesis [41]. More evidence supporting the “decoy” hypothesis is accumulating from studies with phloem-feeding aphids and whiteflies [13], [16], [42] and tissue-damaging herbivores [41]. Thus, we speculate that the induction of “decoy” defense by *P. solenopsis* feeding may have contributed to its rapid invasion in China and elsewhere.

Gossypol is an important allelochemical occurring in glanded cotton varieties, which could be induced by exogenous JA application in cotton [43]. This allelochemical has been demonstrated to be antibiosis to many pests, including phloem-feeding insects. For example, Du et al. [44] found that the high gossypol level in cotton has an antibiotic effect on *Aphis gossypii* in term of aphid longevity and fecundity. Our data show that gossypol level was induced in JA-treated plants where *P. solenopsis* development was delayed. However, gossypol levels were suppressed in SA-treated and *P. solenopsis*-infested plants where *P. solenopsis* showed accelerated development. Thus, we speculate that there is a negative correlation between *P. solenopsis* development and gossypol level, although this needs further experimental verification.

## Supporting Information

Figure S1 Feeding choices of *Phenacoccus solenopsis* females between control leaves (**Control**) and control leaves plus synthetic compounds (**Treatment**). The synthetic compounds and the number of the mealybugs making choices were listed on the right side of the black bars. For each synthetic compound, the absolute amount used for tests was 100 ng. Asterisks represent significant differences from control leaves as determined by replicated *G* test of goodness-of-fit (** P<0.01; n.s. = not significant).
(TIF)

Text S1 (DOC)

## References

[1] Tinsley JD (1898). An ants'-nest coccid from New Mexico.. Can Entomol.

[2] Williams DJ, Granara de Willink MC (1992). Mealybugs of Central and South America..

[3] Abbas G, Arif MJ, Saeed S (2005). Systematic status of a new species of genus *Phenacoccus cockerell* (Pseudococcidae), a serious pest of cotton *Gossypium hirsutum* L., in Pakistan.. Pakistan Entomol.

[4] Yousuf M, Tayyib M, Shazia S (2007). Mealybug problem on cotton in Pakistan.. Pakistan Entomol.

[5] Hodgson C, Abbas G, Arif MJ, Saeed S, Karar H (2008). *Phenacoccus solenopsis* Tinsley (Sternorrhyncha: Coccoidea: Pseudococcidae), an invasive mealybug damaging cotton in Pakistan and India, with a discussion on seasonal morphological variation.. Zootaxa.

[6] Charleston K, Murray D (2010). Exotic mealybug species – a major new pest in cotton.. http://thebeatsheet.com.au/mealybugs.

[7] Wu SA, Zhang RZ (2009). A new invasive pest, *Phenacoccus solenopsis* threatening seriously to cotton production.. Chinese Bull Entomol.

[8] Dutt U (2007). Mealy bug infestation in Punjab: Bt. cotton falls flat.. http://www.countercurrents.org/dutt210807.htm.

[9] Muhammad A (2007). Mealybug: cotton crop's worst catastrophe' published by the Centre for Agro-Informatics Research (CAIR), Pakistan in October 2007.. http://agroict.org/adss/MealyBug_Report.aspx.

[10] Nagrare VS, Kranthi S, Biradar VK, Zade NN (2009). Widespread infestation of the exotic mealybug species, *Phenacoccus solenopsis* (Tinsley) (Hemiptera: Pseudococcidae), on cotton in India.. Bull Entomol Res.

[11] Wang YP, Wu SA, Zhang RZ (2009). Pest risk analysis of a new invasive pest, *Phenacoccus solenopsis*, to China.. Chinese Bull Entomol.

[12] Wang YP, Watson GW, Zhang RZ (2010). The potential distribution of an invasive mealybug *Phenacoccus solenopsis* and its threat to cotton in Asia.. Agr Forest Entomol.

[13] Moran PJ, Thompson GA (2001). Molecular responses to aphid feeding in Arabidopsis in relation to plant defense pathways.. Plant Physiol.

[14] Zhu-Salzman K, Salzman RA, Ahn JE, Koiwa H (2004). Transcriptional regulation of sorghum defense determinants against a phloem-feeding aphid.. Plant Physiol.

[15] Kempema LA, Cui XP, Holzer FM, Walling LL (2007). Arabidopsis transcriptome changes in response to phloem-feeding silverleaf whitefly nymphs. Similarities and distinctions in responses to aphids.. Plant Physiol.

[16] Zarate SI, Kempema LA, Walling LL (2007). Silverleaf whitefly induces salicylic acid defenses and suppresses effectual jasmonic acid defenses.. Plant Physiol.

[17] Li Y, Zou J, Li M, Bilgin DD, Vodkin LO (2008). Soybean defense responses to the soybean aphid.. New Phytol.

[18] Bell E, Creelman RA, Mullet JE (1995). A chloroplast lipoxygenase is required for wound-induced jasmonic acid accumulation in Arabidopsis.. Proc Natl Acad Sci USA.

[19] Fidantsef AL, Stout MJ, Thaler JS, Duffey SS, Bostock RM (1999). Signal interactions in pathogen and insect attack: expression of lipoxygenase, proteinase inhibitor II, and pathogenesis-related protein P4 in the tomato, *Lycopersicon esculentum*.. Physiol Mol Plant Pathol.

[20] Mohase L, van der Westhuizen AJ (2002). Salicylic acid is involved in resistance responses in the Russian wheat aphid-wheat interaction.. J Plant Physiol.

[21] Zhang PJ, Zheng SJ, van Loon JJA, Boland W (2009). Whiteflies interfere with indirect plant defense against spider mites in Lima bean.. Proc Natl Acad Sci USA.

[22] Mewis I, Appel HM, Hom A, Raina R, Schultz JC (2005). Major signaling pathways modulate Arabidopsis glucosinolate accumulation and response to both phloem-feeding and chewing insects.. Plant Physiol.

[23] Pegadaraju V, Knepper C, Reese J, Shah J (2005). Premature leaf senescence modulated by the Arabidopsis *PHYTOALEXIN DEFICIENT4* gene is associated with defense against the phloem-feeding green peach aphid.. Plant Physiol.

[24] Li Q, Xie QG, Smith-Becker J, Navarre DA, Kaloshian I (2006). *Mi*-*1*-mediated aphid resistance involves salicylic acid and mitogen-activated protein kinase signaling cascades.. Mol Plant Microbe Interact.

[25] Thaler JS (1999). Induced resistance in agricultural crops: effects of jasmonic acid on herbivory and yield in tomato plants.. Environ Entomol.

[26] Cooper WR, Goggin FL (2005). Effects of jasmonate-induced defenses in tomato on the potato aphid, *Macrosiphum euphorbiae*.. Entomol Exp Appl.

[27] Ellis C, Karafyllidis I, Turner JG (2002). Constitutive activation of jasmonate signaling in an Arabidopsis mutant correlates with enhanced resistance to *Erysiphe cichoracearum*, *Pseudomonas syringae*, and *Myzus persicae*.. Mol Plant Microbe Interact.

[28] Zhang PJ, Shu JP, Fu CX, Zhou Y, Hu Y (2008). Trade-offs between constitutive and induced resistance in wild crucifers shown by a natural, but not an artificial, elicitor.. Oecologia.

[29] Stipanovic RD, Altman DW, Begin DL, Greenblatt GA, Benedict JH (1988). Terpenoid aldehydes in upland cottons: analysis by aniline and HPLC methods.. J Agric Food Chem.

[30] Artico S, Nardeli SM, Brilhante O, Grossi-de-Sa MF, Alves-Ferreira M (2010). Identification and evaluation of new reference genes in *Gossypium hirsutum* for accurate normalization of real-time quantitative RT-PCR data.. BMC Plant Biol.

[31] Livak KJ, Schmittgen TD (2001). Analysis of relative gene expression data using real-time quantitative PCR and the 2^−ΔΔCt^ method.. Methods.

[32] Marmey P, Jalloul A, Alhamdia M, Assigbetse K, Cacas JL (2007). The 9-lipoxygenase *GhLOX1* gene is associated with the hypersensitive reaction of cotton *Gossypium hirsutum* to *Xanthomonas campestris* pv *malvacearum*.. Plant Physiol Bioch.

[33] Hudspeth RL, Hobbs SL, Anderson DM, Grula JW (1996). Characterization and expression of chitinase and 1, 3-β-glucanase genes in cotton.. Plant Mol Bio.

[34] Zhen XH, Li YZ (2004). Ultrastructural changes and location of β-1, 3-glucanase in resistant and susceptible cotton callus cells in response to treatment with toxin of *Verticillium dahliae* and salicylic acid.. J Plant Physiol.

[35] Dicke M, Gols R, Ludeking D, Posthumus MA (1999). Jasmonic acid and herbivory differentially induce carnivore-attracting plant volatiles in lima bean plants.. J Chem Ecol.

[36] Thaler JS (1999). Jasmonate-inducible plant defences cause increased parasitism of herbivores.. Nature.

[37] Bruinsma M, Van Dam NM, Van Loon JJA, Dicke M (2007). Jasmonic acid-induced changes in *Brassica oleracea* affect oviposition preference of two specialist herbivores.. J Chem Ecol.

[38] Martinez de Ilarduya O, Xie Q, Kaloshian I (2003). Aphid-induced defense responses in *Mi*-*1*-mediated compatible and incompatible tomato interactions.. Mol Plant Microbe Interact.

[39] Pieterse CMJ, Leon-Reyes A, Van der Ent S, Van Wees SCM (2009). Networking by small-molecule hormones in plant immunity.. Nat Chem Biol.

[40] Walling LL (2000). The myriad plant responses to herbivores.. J Plant Growth Regul.

[41] Zhu-Salzman K, Bi JL, Liu TX (2005). Molecular strategies of plant defense and insect counter-defense.. Insect Sci.

[42] Thompson GA, Goggin FL (2006). Transcriptomics and functional genomics of plant defence induction by phloem-feeding insects.. J Exp Bot.

[43] Opitz S, Kunert G, Gershenzon J (2008). Increased terpenoid accumulation in cotton (*Gossypium hirsutum*) foliage is general wound response.. J Chem Ecol.

[44] Du L, Ge F, Zhu SR, Parajulee MN (2004). Effect of cotton cultivar on development and reproduction of *Aphis gossypii* (Homoptera: Aphididae) and its predator *Propylaea japonica* (Coleoptera: Coccinellidae).. J Econ Entomol.

