# Supplementary figures and images for: Suppression of Jasmonic Acid-Dependent Defense in Cotton Plant by the Mealybug Phenacoccus solenopsis
Source: PLoS One. 2011 Jul 27;6(7):e22378. doi: 10.1371/journal.pone.0022378 (PMC3144893; doi:10.1371/journal.pone.0022378)

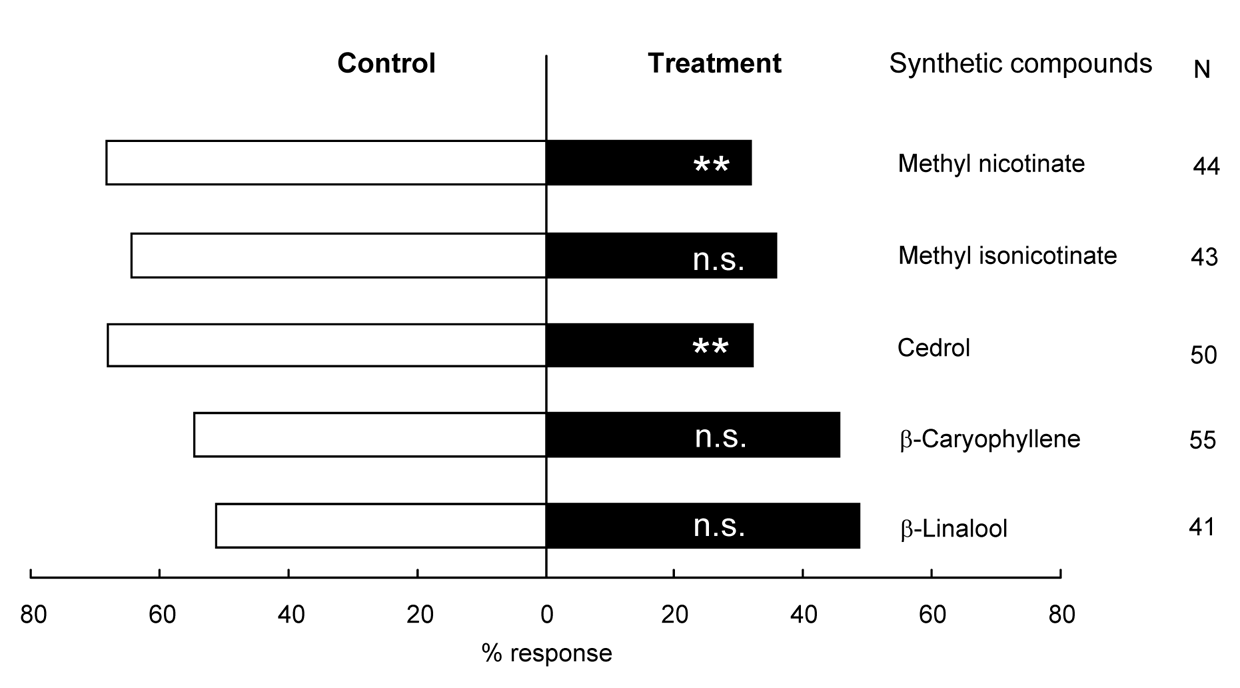

Supplement: Figure S1 — Feeding choices of Phenacoccus solenopsis females between control leaves (Control) and control leaves plus synthetic compounds (Treatment). The synthetic compounds and the number of the mealybugs making choices were listed on the right side of the black bars. For each synthetic compound, the absolute amount used for tests was 100 ng. Asterisks represent significant differences from control leaves as determined by replicated G test of goodness-of-fit (** P<0.01; n.s. = not significant). (TIF) [file pone.0022378.s001.tif]
