# Supplementary material for: Suppression of Jasmonic Acid-Dependent Defense in Cotton Plant by the Mealybug Phenacoccus solenopsis
Source: PLoS One. 2011 Jul 27;6(7):e22378. doi: 10.1371/journal.pone.0022378 (PMC3144893; doi:10.1371/journal.pone.0022378)
Supplement: Text S1 — (DOC) [file pone.0022378.s002.doc]

**Supporting Information to:**

**Suppression of jasmonic acid-dependent defense in cotton plant by the mealybug *Phenacoccus solenopsis***

Pengjun Zhang^1*^, Xiaoyun Zhu^1, 2^, Fang Huang^1^, Yong Liu^1^, Jinming Zhang^1^, Yaobin Lu^1*^, Yongming Ruan^3^

^1^ State Key Laboratory Breeding Base for Zhejiang Sustainable Pest and Disease Control; Institute of Plant Protection and Microbiology, Zhejiang Academy of Agricultural Sciences, Hangzhou 310021, China

^2^ Department of Plant Protection, Nanjing Agriculture University, Nanjing 210095, China

^3^ College of Chemistry and Life Sciences, Zhejiang Normal University, Jinhua 321004, China

**Materials and Methods**

**Feeding choice tests with synthetic compounds.**

Methyl isonicotinate, methyl nicotinate, cedrol, β-Linalool and β-caryophyllene (Sigma-Aldrich) were diluted in n-hexane (Sigma-Aldrich, HPLC grade) to gain a solution with dose of 100 ng⋅μl^-1^, respectively. In all experiments, 1μl of the diluted solution or hexane was applied on a piece of cotton wool (0.5 cm^2^). In this experiment, mealybugs were offered a choice of two detached leaves from undamaged plants: one leaf plus a cotton wool containing hexane was regarded as control, and another leaf plus a cotton wool containing one of synthetic compounds regarded as treatment. Two leaves were placed opposite each other in a Petri dish (diam 14.5 cm) covered with moist filter paper, so that they were approximately 5 cm apart at the closest point. The position of the leaves was alternated between replicates. Immediately, four new-emerged adult female mealybugs were transferred into each dish in the gap between the leaves. After 12 h, the number of adults on each of the two leaves was counted. The experiments were repeated for 10-15 times for each treatment.

**Statistical Analysis**

A replicated *G*-test of goodness-of-fit was used to analyze the feeding choice of *P. solenopsis* between treated and control leaves, with the null hypothesis of no preference. Females that did not make a choice were excluded from the analysis.

**Results and Discussion**

After adding methyl nicotinate to leaves, as much as 68.2% of *P. solenopsis* females preferred the control leaves (*P* = 0.01; Figure S1), indicating a repellency effect of methyl nicotinate. Cedrol has a similar effect: after adding cedrol to leaves, 68% of *P. solenopsis* females preferred the control leaves (*P* = 0.01; Figure S1). In contrast, after adding methyl isonicotinate, β-caryophyllene, or β-linalool to leaves, *P. solenopsis* females did not discriminate between control leaves and control leaves plus these synthetic compounds (Figure S1).

Of the five synthetic compounds, methyl nicotinate, cedrol, and methyl isonicotinate were significantly induced in JA-treated plants. Thus, the repellency effect caused by methyl nicotinate and cedrol suggested that the volatiles emitted from JA-treated plants played an important role in repelling *P. solenopsis* females.

β-linalool and β-caryophyllene were two major components of the volatiles emitted from mealybug-infested plants. *P. solenopsis* females showed a preference to the mealybug-infested plants. Since these two compounds were not attractive for *P. solenopsis* females, we speculated that one or more of other volatile compounds, or their combinations, could play an important role in attracting mealybugs. These need further experiments for verification.
